# Continuous secretory production in *E. coli* enables scalable, high-titer manufacturing of active recombinant endonucleases

**DOI:** 10.1186/s13036-025-00590-0

**Published:** 2025-12-15

**Authors:** Sudarsana Reddy Lokireddy, Chennakesavulu Thummadhi, Pratyusha Godavarty, Venkateswarlu Petla, Akhila Munimanda, Sridhar Rao Kunchala, Ramakrishna Vadde

**Affiliations:** 1 Oncosimis Biotech Private Limited, Plot No 3, Genpact Rd, IDA Uppal, Hyderabad, Telangana 500007 India; 2 Department of Biotechnology and Bioinformatics, Yogi Vemana University, Kadapa, Andhra Pradesh 516005 India

**Keywords:** *Escherichia coli*, Protein secretion, Signal peptide, Recombinant proteins, Endonuclease, DRNase^®^ and DNase I

## Abstract

Recombinant endonucleases are essential for biopharmaceutical manufacturing and molecular biology workflows, yet their intracellular expression in *Escherichia coli* often leads to host cell toxicity due to non-specific DNA and RNA degradation. To address this, we employed the BacSec^®^ system to secrete *Serratia marcescens* non-specific endonuclease, nucA (DRNase^®^) and bovine DNase I in *E. coli*, aiming to mitigate cytotoxicity and streamlined downstream processing. DRNase^®^ was efficiently secreted, enabling simplified purification at the shake flask level and achieving 1 g/L in high-density fermentation, with over 2 g/L in perfusion-based fermentation. The secreted DRNase^®^ was predominantly monomeric, demonstrated higher specific activity than commercial counterparts, and remained stable at room temperature for over a year. Likewise, secreted bovine DNase I retained strong enzymatic activity without degrading mRNA, making it particularly suitable for mRNA vaccine production. These secreted endonucleases support a wide range of industrial applications, including biologics production, gene therapy, mRNA and viral vector-based vaccines, and therapeutic use. Overall, the BacSec^®^ platform, integrated with perfusion fermentation, provides a scalable, tag-free, and cost-effective solution for high-titer production of active endonucleases.

**Supplementary Information:**

The online version contains supplementary material available at 10.1186/s13036-025-00590-0.

## Background

Historically, recombinant proteins expressed in *E. coli* accumulate in the cytoplasm, which often results in the formation of inclusion bodies (insoluble aggregates of misfolded proteins) that are often cytotoxic to the host cells [1, 2]. Although proteins from inclusion bodies can be recovered through solubilization and refolding processes, these steps introduce added complexity and cost, and frequently lead to reduced yiels and compromised biological activity [1, 3]. Furthermore, intracellular expression imposes challenges for downstream processing, particularly at commercial scales, due to the need for mechanical cell disruption and separation of the target protein from abundant host-derived contaminants [2, 3]. To overcome these limitations, secretory expression systems have been developed in *E. coli* to facilitate the export of recombinant proteins either into the periplasmic space or directly into the extracellular medium [4–9]. Multiple strategies have been explored to enable or enhance extracellular secretion in *E. coli* [5, 7, 10]. One common method involves secretion into the periplasm followed by chemical, enzymatic, or physical treatments to increase outer membrane permeability, thereby releasing the target protein [11–14]. However, this approach can damage the protein and is generally unsuitable for large-scale processes. Another method involves the use of L-form *E. coli* strains that lack an outer membrane, allowing proteins exported through the inner membrane to appear directly in the culture medium [14, 15]. Despite this advantage, these strains exhibit poor growth and instability, making them unsuitable for industrial applications. Further attempts have been made to utilize and engineer native secretion systems, such as fusing specific export signals to the target protein and co-expressing essential translocation machinery [4, 16, 17].

While some success has been achieved, overall protein titers have remained modest and few systems have reached commercial viability [18]. Systems like the hemolysin A (HlyA)-based T1SS [9], and recent innovations such as BacSec^®^ technology [4], and ESETEC^®^ [18] are examples of how targeted secretion systems are transforming *E. coli* into an efficient and scalable cell factory for secretory protein production. BacSec^®^ and ESETEC^®^ are innovative secretion technology that enables high-level extracellular expression of recombinant proteins in *E. coli* [4, 18]. BacSec^®^ is designed to overcome the limitations of traditional secretion systems by minimizing contaminants such as host cell protein, endotoxins, and nucleic acids, thereby simplifying purification processes, reducing production time, and lowering manufacturing costs [4]. Notably, this platform allows for the production of proteins that are difficult to express due to the toxicity or misfolding in the cytoplasm, providing a robust solution for challenging targets [18].

Among the many valuable proteins that benefit from secretory production systems are endonucleases that catalyze the internal cleavage of phosphodiester bonds in nucleic acids [19]. Endonucleases (*Serratia* nuclease and DNase I) are indispensable tools in biotechnology and medicine, used for removing residual host DNA and RNA during biologics production, gene therapy, mRNA vaccines, viral vector-based vaccines, cell dissociation reagents in diagnostic assays, and enabling genome editing techniques [20–24]. Moreover, these nucleases hold significant potential for therapeutic applications, including in respiratory diseases and chronic wound healing, and have been reported to possess medical importance [25–28]. However, these enzymes are quite expensive for large scale biologics preparations due to complex production process.

This study demonstrates the utility of the BacSec^®^ platform for the extracellular production of high-quality, monomeric, and RNase-free DRNase^®^ (similar to Benzonase^®^) and DNase I in *E. coli*. By directing secretion into the culture medium, BacSec^®^ platform technology eliminates the need for cell lysis, solubilization, and refolding, significantly reducing downstream complexity and processing costs. The platform ensures high titer, purity, and enzymatic activity combined with use of a chemically defined medium we achieved a greater batch-to-batch consistency and subsequent regulatory compliance. When integrated with continuous or perfusion-based fermentation strategies, BacSec^®^ technology further enhances productivity and reduces carbon footprint. These advantages position BacSec^®^ as a scalable and industrially viable solution for the production of sensitive or high-value proteins such as bioprocess-grade DRNase^®^ and DNase I at affordable cost.

## Results

### Shaker flask-level expression and secretion of nuclease A (nucA) (DRNase^®^) in *E. coli*

**Fig. 1:**
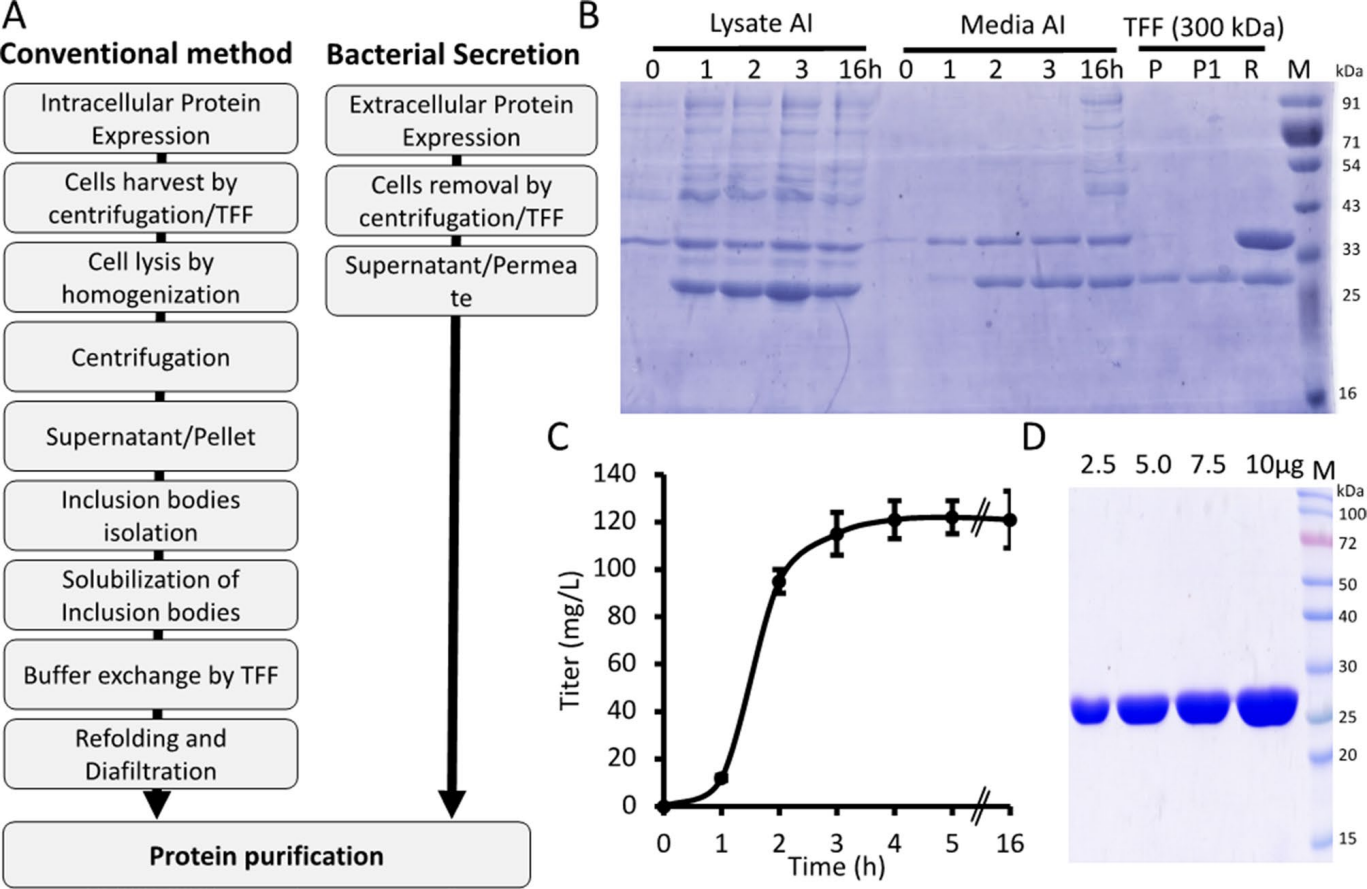
Shaker flask-level expression and secretion of Nuclease A (nucA) (DRNase^®^) in *E. coli.* **A**. Schematic representation of industrial-scale recombinant protein production via conventional intracellular expression versus BacSec^®^-mediated bacterial secretion. **B**. Time-dependent expression and secretion of DRNase^®^ in shaker flasks, with corresponding SDS-PAGE analysis of TFF-processed culture supernatants (P- Permeate; P1-Flux permeate; R-Retentate). 10X concentrated retentate was loaded on SDS-PAGE to show contaminated protein (thicker band). 5 µl of media samples were loaded in each well. **C**. Quantification of secreted DRNase^®^ in the culture medium at various time intervals by ELISA. Data are shown as mean ± SEM from triplicates of the same culture medium. **D**. SDS-PAGE (12%) analysis of increasing enzyme concentrations (2.5–10 µg/well) demonstrated the purity of DRNase^®^ following anion exchange chromatography. AI = After Induction

Expressing endonucleases as secretory proteins in *E. coli* preserves native folding and functional activity while simplifying downstream purification. This approach offers clear advantages over conventional methods such as inclusion body formation, including higher purity, improved lot-to-lot consistency, and better scalability (Fig. 1A). Secretion into the extracellular medium also minimizes degradation by intracellular proteases and enables efficient recovery of active enzyme in a cost-effective manner (Fig. 1A). To identify the optimal signal peptide for secretion, the *E. coli* codon-optimized *nucA* gene was synthetically constructed with different signal peptides (*yefB*, modified *yefB*, *LPP*, *pelB*, *ompA*, and *ompF*) (Table S1) and cloned into the pBacSec plasmid, which contains a kanamycin resistance marker and an IPTG-inducible promoter (Fig. S1A). The secretion-optimized *E. coli* strain BOP2 (complete genomic characterization of the BOP2 strain under investigation*)* were transformed with pBacSec vector containing gene of interest and the colonies expressing each construct were grown in 3 mL of chemically defined media with IPTG inducer. The extracellular secretion of protein was confirmed by SDS-PAGE (Fig. S1A). Among the signal peptides tested, pelB signal peptide directed the highest level of DRNase^®^ secretion with comparably less intracellular accumulation was selected for further experiments (Fig. S1A).

At the production flask scale (1 L), extracellular secretion of DRNase^®^ was monitored post-IPTG induction by sampling the cultured media at various time points and analyzing it by SDS-PAGE and ELISA (Fig. 1B-C). DRNase^®^ secretion increased linearly up to 3 h post-induction; however, overnight induction adversely affected protein produced due to bacterial lysis (Fig. 1B-C). After three hours of induction, culture media was clarified by centrifugation at 15,000 × *g* for 15 min. The clarified cultured media was subjected to Tangential Flow Filtration (TFF) using a 300 kDa hollow fiber membrane to collect the permeate. The collected permeate was then dialyzed in 25 mM Tris (pH 8.0) using a 10 kDa TFF cassette. SDS-PAGE analysis of the retentate revealed that the secreted DRNase^®^ was over 90% pure (Fig. 1B). The 300 kDa retentate (10 times concentrated) contained two major bands that corresponded to flagellin and aggregated DRNase^®^ (Fig. 1B). The 10 kDa dialyzed retentate was further purified using a 50 mL Q-Sepharose column and eluted with a linear salt gradient (Fig. S1B). Final SDS-PAGE analysis with various concentration of protein revealed a single band corresponding to DRNase^®^, indicating > 99% purity (Fig. 1D). Moreover, the endotoxin levels quantified by recombinant Factor-C endotoxin detection kit and also confirmed LAL assay (gel clot assay) were found to be < 0.5EU/mg of Protein (data not shown).

### DRNase^®^ exhibits higher activity compared to commercially available endonuclease

**Fig. 2:**
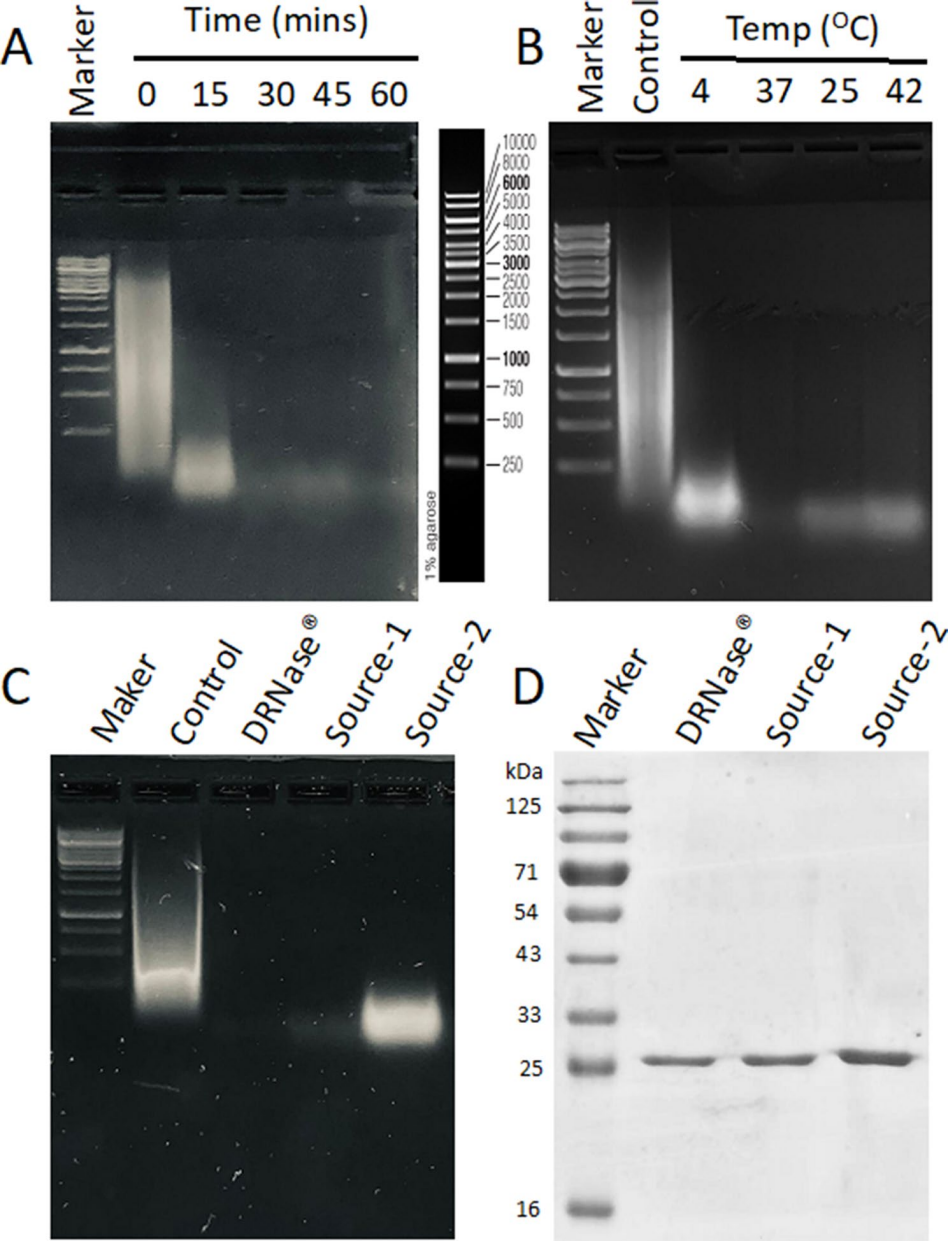
DRNase^®^ exhibits superior activity compared to commercially available endonuclease. **A & B.** Digestion of 50 µg sonicated salmon sperm DNA with 1 unit of DRNase^®^ in 25 mM Tris-HCl buffer (pH 8.0) containing 20 mM NaCl and 2 mM MgCl₂, assessed in a time- and temperature-dependent manner. Reaction products were analyzed on a 1% agarose gel. **C.** Comparative digestion of 50 µg salmon sperm DNA using 1 unit each of DRNase^®^ and two commercially available similar endonucleases for 30 min. The enzymatic activity, as visualized on a 1% agarose gel, was found to be higher than that of the competitor enzymes. **D.** SDS-PAGE (12%) analysis of DRNase^®^ and the two commercial DNase I enzymes using equivalent enzymatic units to assess purity and molecular weight. Commercially purchased endonucleases from Supplier 1 (Source-1) and Supplier 2 (Source-1)

The purified DRNase^®^ was evaluated for its enzymatic activity using the acid-soluble nucleotide assay following the standard protocol. One unit of activity is defined as the amount of enzyme required to digest sonicated salmon sperm DNA to acid-soluble oligonucleotides equivalent to a ΔA260 of 1.0 in 30 min at pH 8.0 and 37 °C in a reaction buffer. DRNase^®^ exhibited an activity of 1.5–1.6 × 10⁶ units per milligram of protein. Further characterization was performed to assess stability under various conditions, including different incubation times, temperatures, pH values, freeze-thaw cycles, and protease activity. Interestingly, DRNase^®^ has shown a reasonable activity at 4 °C, although it was less as compared to its activity at 25 °C and 37 °C (Fig. 2B). DRNase^®^ exhibits optimal activity in the range of pH 7–9 (Fig. S2A). To evaluate stability under stress conditions, the enzyme was subjected to 15 freeze-thaw cycles. Remarkably, DRNase^®^ maintained its activity even after 15 cycles (Fig. S2B). No protease activity was observed when tested with BSA as a substrate, confirming its specificity (Fig. S2C).

To assess long-term stability, DRNase^®^ was stored at -20 °C, 4 °C, and room temperature (24 °C) and monitored its activity over a period of one year. The DRNase^®^ enzyme retained full activity under all storage conditions, indicating excellent stability (Fig. S2D). In comparative assays, DRNase^®^ exhibited higher endonuclease activity compared to commercially available alternatives (source-1 and source-2) (Fig. 2C and Fig. S2E). Further analysis using SDS-PAGE followed by densitometric quantification revealed that DRNase^®^ is present in a lower total protein amount compared to the commercially available enzymes (Fig. 2D and Fig. S2E). In conclusion, the secreted DRNase^®^ demonstrated significantly enhanced specific activity and stable catalytic efficiency across a range of temperatures, underscoring its potential as a robust and efficient tool for DNA degradation in biotechnological and industrial applications.

### Secreted DRNase^®^ exists predominantly in monomeric form

**Fig. 3:**
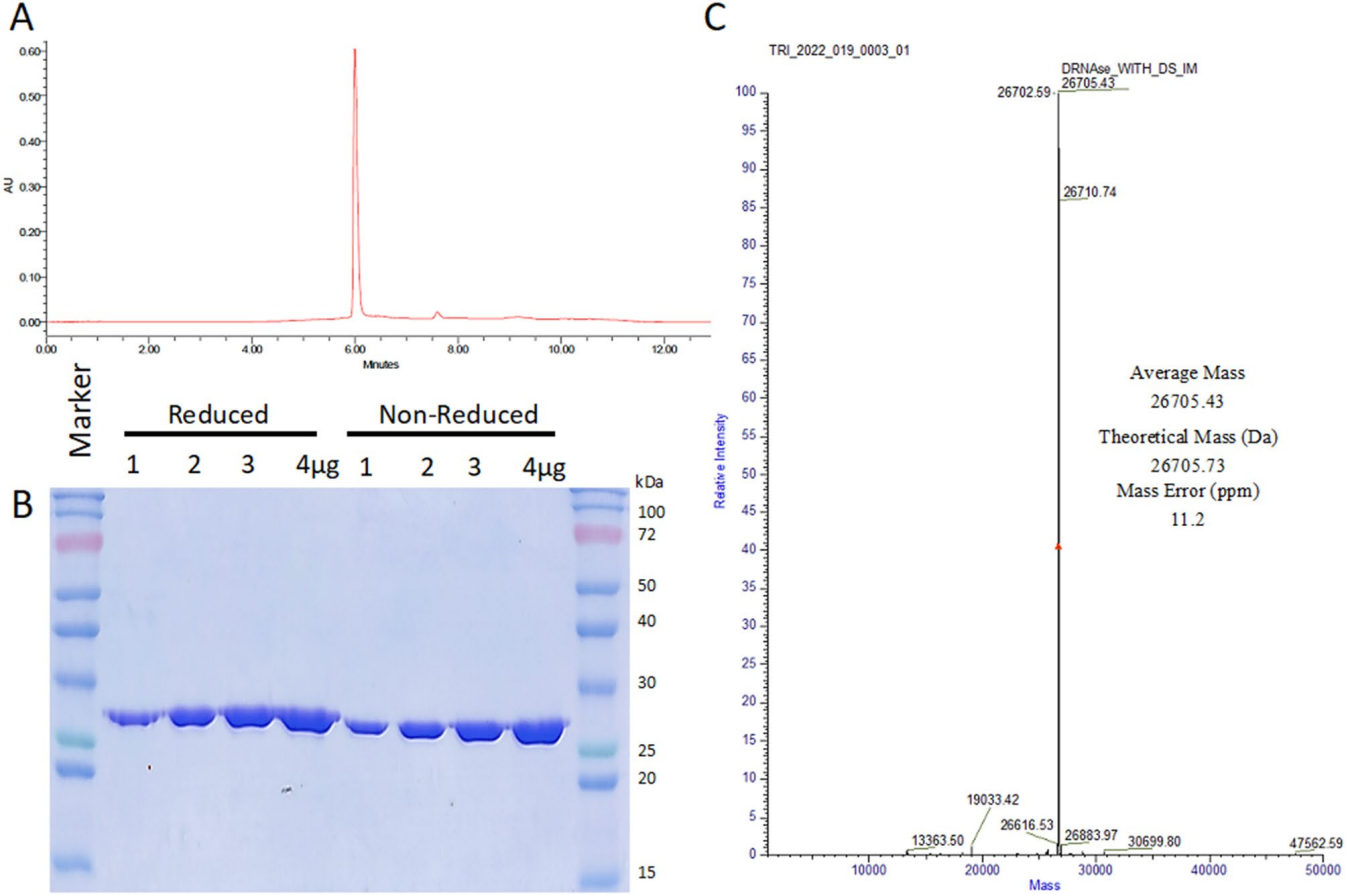
Secreted DRNase^®^ exists predominantly in monomeric form. **(A)** UPLC chromatogram showing the purity of DRNase^®^ after separation on a C18 column under gradient elution. **(B)** Increasing concentration of purified DRNase^®^ assessed under reducing (heated with DTT) and non-reducing (unheated, without DTT) conditions by SDS-PAGE analysis confirming monomeric form. **(C)** Intact mass analysis of DRNase^®^ using UHPLC-UV-MS, displaying the molecular weight of profile and confirming monomeric structure (26705.43 Da with mass error of 11.2 ppm)

To assess the purity of the protein, we performed UPLC using a C18 column. The analysis showed a single peak, indicating >99% purity (Fig. 3A). The same protein sample was analyzed by SDS-PAGE under both reducing and non-reducing conditions. DRNase^®^ displayed an identical molecular weight in both conditions, suggesting it exists as a monomer (Fig. 3B). To further confirm the molecular characteristics, intact mass analysis was performed using UHPLC-UV-MS. Consistent with the SDS-PAGE results, the intact mass of DRNase^®^ was determined to be 26,705.43 Da, closely matching the theoretical mass of 26,705.73 Da, with a mass error of only 11.2 ppm (Fig. 3C and Fig. S3A-B). Collectively, these data confirms that Secreted DRNase^®^ indeed exist as monomeric form. However, commercially available counterpart, Benzonase^®^ exists as homodimer [20, 21, 29, 30].

Tertiary structure analysis was performed using circular dichroism (CD) spectroscopy. Far-UV CD spectra recorded at 25 °C revealed that DRNase^®^ consists of 31.9% α-helix, 13.7% β-sheet, 14.5% turns, and 39.9% other structures (Fig. S4A). Thermal structural stability was assessed using near-UV CD spectra at temperatures ranging from 25 °C to 75 °C, in 5 °C increments. Structural changes were minimal up to 40 °C; however, a distinct shift in secondary structure was observed at 45 °C and above, with a complete loss of structure at higher temperatures, as indicated by a flattened CD spectrum (Fig. S4B).

### High-density fermentation for the production of DRNase^®^

**Fig. 4:**
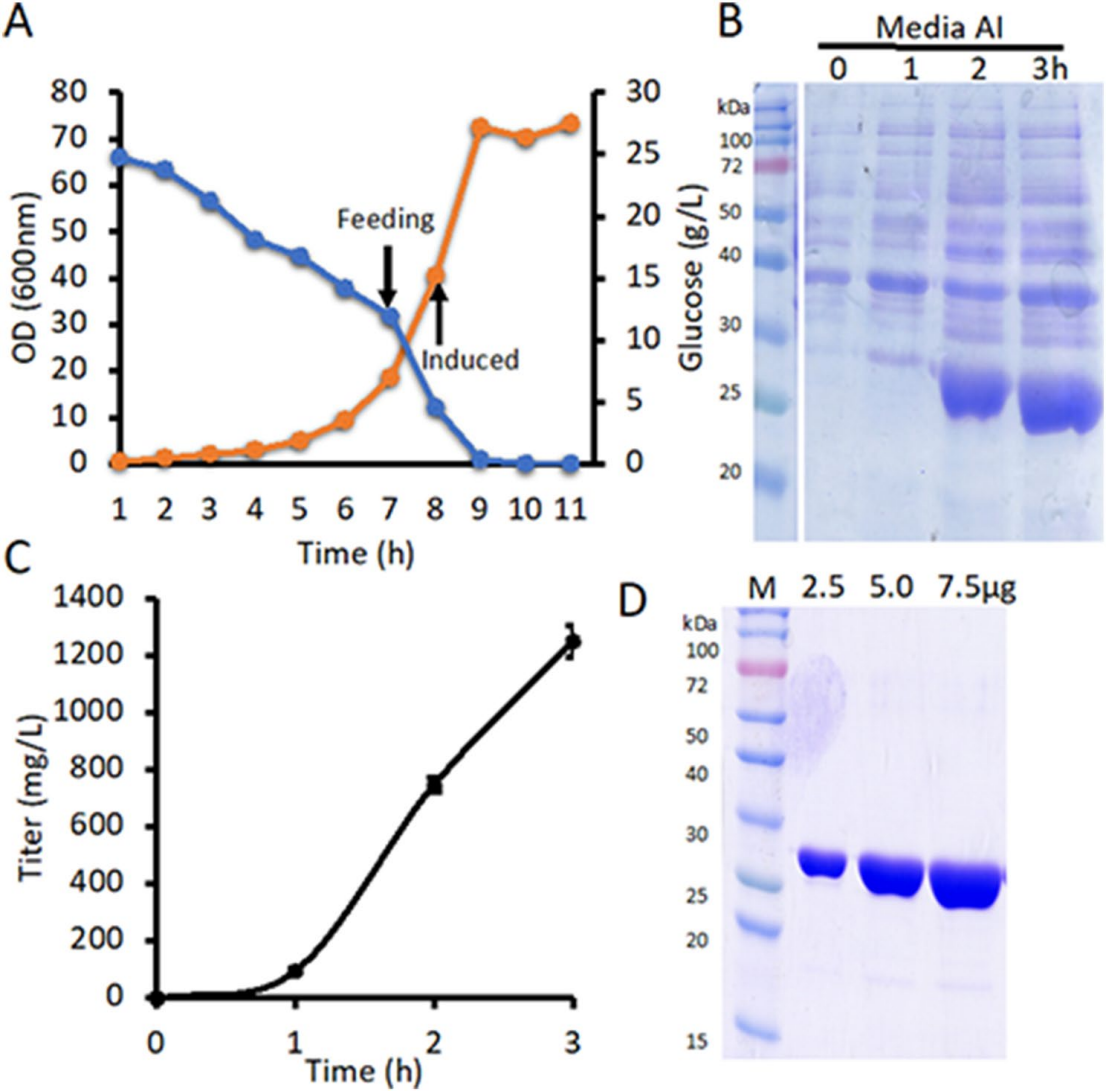
High-density fermentation for the production of DRNase^®^. **(A)** Line graph showing cell growth (OD₆₀₀) (orange) and glucose (blue) concentration during fermentation. The arrow indicates the point of feed initiation and IPTG induction. **(B)** Time-dependent expression and secretion of DRNase^®^ in the fermenter, analyzed by SDS-PAGE. 5 µl of media samples were loaded in each well. **(C)** Quantification of secreted DRNase^®^ in the culture supernatant at different time intervals using ELISA. Data are shown as mean ± SEM from triplicates of the same culture medium. **(D)** SDS-PAGE analysis showing the purity of DRNase^®^ after anion exchange chromatography. AI = After Induction

To further enhance titer, DRNase^®^ production was scaled up using a high-cell-density fed-batch fermentation process in a 3 L Minifors lab-scale fermenter. An overnight seed culture was inoculated at 10% (v/v) into fresh chemically defined medium containing 2.5% glucose. Fermentation was conducted at 37 °C under cascade control mode (stirring > air > oxygen) with pH maintained at 6.85 and dissolved oxygen (DO) set at 30%. Bacterial growth and glucose levels were monitored hourly. The culture doubled approximately every hour, and upon glucose depletion to ~ 10 g/L, a feeding strategy was initiated to promote further biomass accumulation. When the optical density (OD₆₀₀) reached 40, with glucose maintained at ~ 10 g/L, the culture was induced with 0.25 mM IPTG (Fig. 4A). Post-induction, samples were collected hourly to monitor OD and protein expression. The fermentation was terminated after 3 h of induction. Secretion of DRNase^®^ was confirmed by ELISA and SDS-PAGE (Fig. 4B-C), with ELISA quantification indicating an approximate titer of 1250 mg/L in the culture media before purification (Fig. 4C). The DRNase^®^ was purified using the same protocol as described for shaker-flask cultures, resulting in > 99% purity under high-density fermentation conditions as analyzed by SDS-PAGE (Fig. 4D). We achieved a titer of 975 mg/L produced after the final purification with a specific activity of 1.5 × 10⁶ units/mg of protein.

### Production of DRNase^®^ in perfusion-based fermentation method

**Fig. 5:**
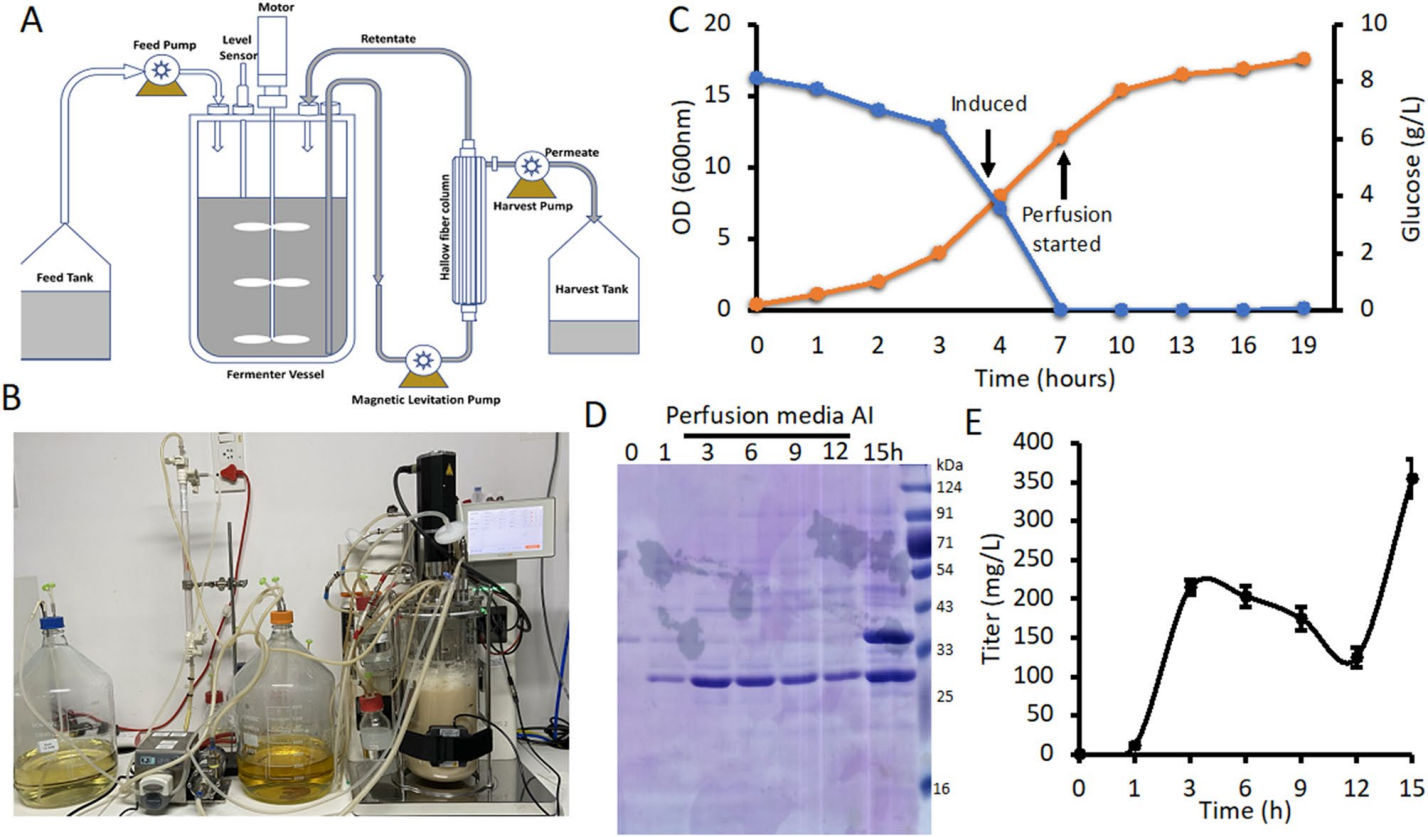
Production of DRNase^®^ in perfusion-based fermentation method. **A & B.** Pictorial representations illustrating the setup and workflow of perfusion-based fermentation. **C.** Line graph depicting cell growth (OD₆₀₀) (orange line) and glucose (blue line) concentration over time during fermentation. The arrow marks the initiation of feeding and IPTG induction. **D.** SDS-PAGE analysis showing time-dependent expression and secretion of DRNase^®^ during and post-perfusion phase. 5 µl of media samples were loaded in each well. **E.** ELISA-based quantification of DRNase^®^ levels in the culture supernatant at various time points. Data are shown as mean ± SEM from triplicates of the same culture medium. AI = After Induction

The BacSec^®^ platform enables the secretion of recombinant proteins directly into the extracellular medium from *E. coli*, mimicking the secretion profile similar to that of mammalian cell culture-based platform [31]. To enhance titer and maintain cell viability over extended time period, we implemented a perfusion-based fermentation strategy as described above. Three hours post-induction with IPTG, continuous perfusion was initiated by passing culture media through a hollow fiber perfusion module (300 kDa MWCO, 1000 cm² surface area). Collecting the permeate containing DRNase^®^ and simultaneously feeding of the culture with fresh medium. The system was configured such that the culture broth was circulated through the module via a single inlet to the bioreactor, enabling real-time separation of the product-containing permeate while returning cells back to the vessel (Fig. 5A-C). Perfusion was operated at a rate of 1 L/hour using synchronized peristaltic pumps. Every 3 h, an equal volume of fresh medium was added, allowing for continuous nutrient replenishment and removal of spent media. The perfusion system-comprising a diaphragm pump, MW-specific hollow fiber membrane, filtrate line, and control unit-ensured minimal cell residence time outside the fermenter, preserving cell viability and optimizing protein secretion (Fig. 5A-B). This system is run about 19 h with simultaneous collection of the product (Fig. 5C).

This strategy significantly enhanced productivity. From a 3 L fermentation run, a total of 15 L of permeate was collected, titer an average DRNase^®^ concentration of 200 mg/L in the perfusate. This corresponds to a total secreted titer of ~ 2 g/L, resulting in 5.2 g of highly purified DRNase^®^ with a specific activity of 1.5 × 10⁶ units/mg (Fig. 5D-E). Post 12 h induction, there was a decrease in secretion over the time could be due to exhaustion of *E. coli* for protein expression and secretion (Fig. 5D-E). Interestingly, a 35 kDa contaminant was consistently retained in the retentate and did not pass through the 300 kDa hollow fiber membrane. This protein is hypothesized to be multimeric flagellin, which may exceed the membrane cut-off due to its quaternary structure (Fig. 5D). Overall, the protein production by perfusion-based fermentation system offers a cost-effective and scalable alternative to fed-batch method, especially when paired with the BacSec^®^ secretion platform. The use of inexpensive, chemically defined medium, combined with sustained cell viability and high secretion efficiency, positions this approach as a robust method for large-scale production of extracellular recombinant proteins.

### Recombinant RNase-free DNase I was efficiently secreted by *E. coli*

**Fig. 6:**
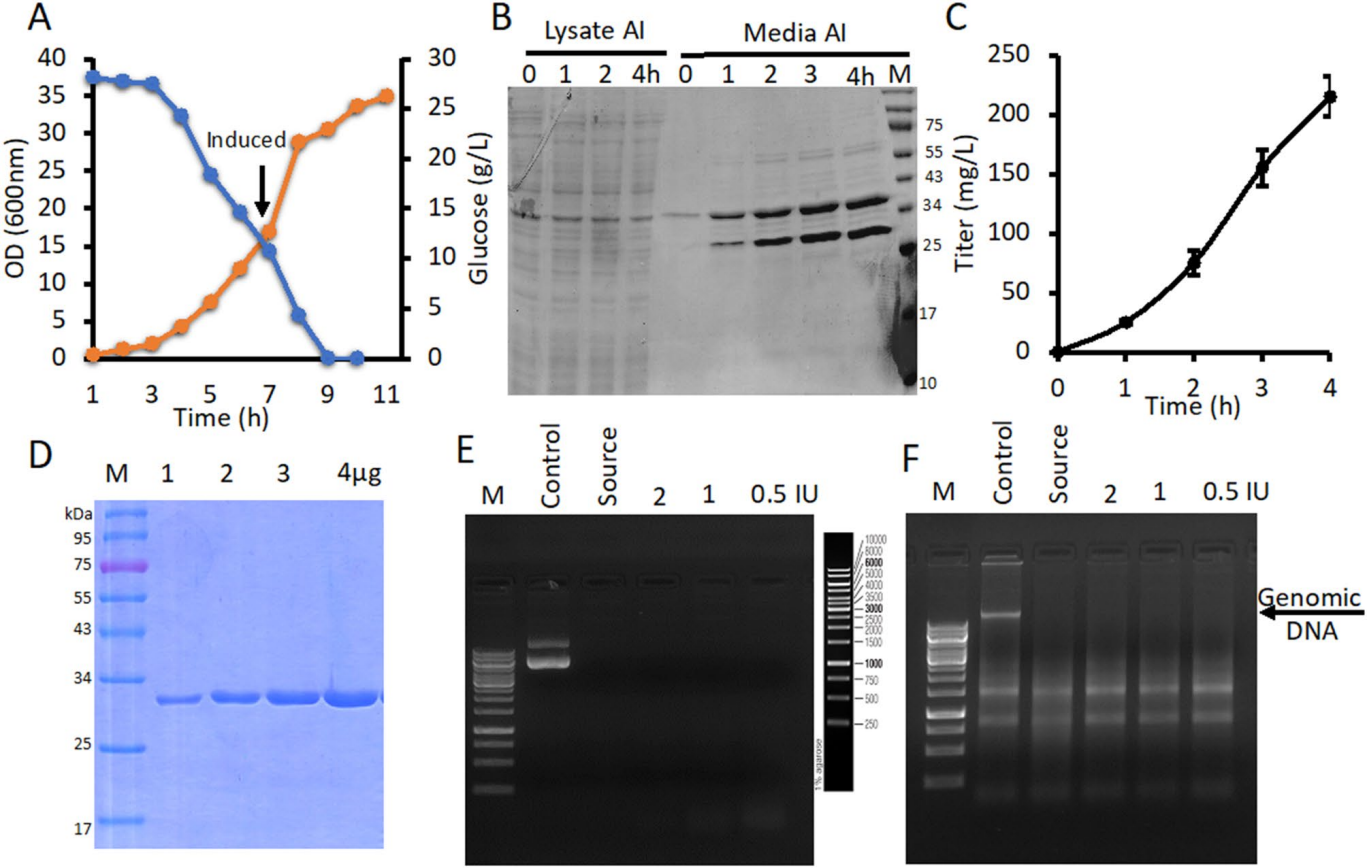
Recombinant RNase-free DNase I was efficiently secreted by *E. coli*. **(A)** Line graph showing cell growth (OD₆₀₀) (orange line) and glucose (blue line) concentration over time during fermentation. The arrow indicates the point of IPTG induction. **(B)** SDS-PAGE analysis of time-dependent expression and secretion of DNase I following induction. 10 µl of media samples were loaded in each well. **(C)** ELISA-based quantification of secreted DNase I in the culture supernatant at various time intervals. Data are shown as mean ± SEM from triplicates of the same culture medium. **(D)** SDS-PAGE analysis demonstrating the purity of DNase I after Ni-NTA affinity chromatography. **(E)** Digestion of 1 µg plasmid DNA with 1 unit of DNase I in 10 mM Tris-HCl (pH 7.5), 2.5 mM MgCl₂, and 5 mM CaCl₂, compared to a commercial DNase I (Source). Reactions were performed for 10 minutes in a dose-dependent manner and analyzed on a 1% agarose gel.** (F) **The same enzyme dilutions were incubated with genomic DNA contaminated with RNA (2µg). Agarose gel analysis shows complete digestion of genomic DNA, while RNA remained intact. Arrow indicates undigested RNA. AI = After Induction

Another important endonuclease is DNase I, which specifically degrades DNA, unlike the non-specific DRNase^®^ derived from *Serratia marcescens* that degrade all nucleic acids. DNase I holds significant economic potential in mRNA manufacturing, where it is used to degrade residual template DNA while preserving the integrity of the synthesized mRNA. So, next we evaluated the secretion of recombinant DNase I using a similar strategy. A codon-optimized DNase I gene was synthesized with an N-terminal *pelB* signal peptide and cloned into the pBacSec^®^ expression vector to facilitate extracellular secretion. Following transformation, multiple clones were screened, and a lead candidate was selected for high-density fermentation. In a 4-liter fermenter vessel, 300 mL of seed culture was inoculated into 3 L of freshly prepared chemically defined medium supplemented with 2.5% glucose and 3 mL of kanamycin (50 µg/mL). Fermentation was carried out until the culture reached an OD₆₀₀ of 18, with glucose maintained at ~ 10 g/L. Induction was initiated by the addition of 0.25 mM IPTG (Fig. 6A).

Post-induction, culture samples were collected hourly to monitor growth and protein expression. Fermentation was terminated after 4 h of induction. Secretion of DNase I into the culture media was confirmed by SDS-PAGE and ELISA (Fig. 6B-C), with ELISA quantification indicating approximately 250 mg/L of DNase I in the unpurified culture media (Fig. 6C). Purification was performed using the same protocol as for shaker-flask experiments, yield DNase I at > 99% purity (Fig. 6D). The final purified protein titer was approximately 200 mg/L with a specific activity of 6–7 × 10⁴ units/mg. Importantly, the purified enzyme was tested for functional specificity by incubating it with total RNA samples spikes with plasmid DNA (Fig. 6E). DNase I effectively degraded the DNA without affecting RNA integrity, confirming that the preparation was free from RNase activity (Fig. 6F). This makes the secreted DNase I particularly well-suited for applications such as mRNA vaccine manufacturing, where selective removal of DNA templates is critical.

## Discussion

Efficient production of active endonucleases in *E. coli* systems remains challenging due to their inherent cytotoxicity and tendency to form insoluble inclusion bodies. This study addresses these limitations by demonstrating a robust secretion-based expression strategy for DRNase^®^ and DNase I in *Escherichia coli* using the BacSec^®^ system. By directing the target enzyme secreted into the extracellular medium, retaining the native folding and enzymatic activity significantly reducing the cytotoxicity and simplifying the downstream processing. A key aspect of this strategy was the systematic screening of signal peptides to optimize secretion efficiency. Among six candidates, the pelB signal peptide was identified as the most effective, enabling efficient translocation of endonuclease into the extracellular space with minimal intracellular accumulation. This result is consistent with previous findings that highlight pelB versatility in Sec-pathway-dependent protein export, particularly for proteins prone to misfolding in the cytoplasm [32]. The in-house developed secretory *E. coli* strain BOP2, grown in chemically defined media under carefully controlled conditions, enabled high-level secretion of endonucleases with low contaminated proteins. Secretion was peaked approximately three hours post-induction, after which prolonged induction led to increased host cell lysis and accumulation of contaminating proteins, including flagellin. This underscores the importance of tightly timed induction to maximize product titer and maintain culture integrity. Integration of a 300 kDa hollow fiber perfusion module enabled continuous harvesting of the secreted enzyme, resulting in a total of 15 L of permeate from a 3 L culture and a final produced increased to 2-fold compared to fed-batch method [33]. This level of volumetric productivity and overall recovery is rarely achieved in bacterial systems, where perfusion processes are often avoided due to foaming, membrane clogging, and oxygen limitations.

DRNase^®^ exhibited more catalytic efficiency compared to commercially available alternatives. The specific activity of the purified DRNase^®^ enzyme was 1.5–1.6 × 10⁶ units/mg, which exceeded that of commercially available other nucleases (1.0-1.2 × 10⁶ units/mg) in DNA degradation assays [21]. Intact mass analysis using UHPLC-UV-MS confirmed a molecular mass of 26,705.43 Da, in close agreement with the theoretical mass of 26,705.73 Da, with a minimal mass error of 11.2 ppm. In contrast to commercially available nuclease which is expressed intracellularly and exists as a homodimer [29, 30], where DRNase^®^ existed monomeric form with higher specific activity. In contrast, it is reported that being a dimer of endonuclease have more advantages, specifically, dimerization leads to a rearrangement of the primary DNA-binding contacts, as the transition from monomer to dimer alters the local electrostatic field compared to monomeric form [34, 35]. It was previously demonstrated that the *Serratia* nuclease variants H184A, H184N, H184T, and H184R exist as monomers and retain secondary structure, stability toward chemical denaturation, and catalytic activity comparable to the wild-type enzyme [35, 36]. These results indicate that the dimeric state is not essential for the catalytic function of *Serratia* nuclease [36]. Despite predominantly existing as a monomer, DRNase^®^ mechanistic basis for this enhanced activity is likely more complex than monomeric state alone, and may involve differences in folding, secretion-associated maturation, or other structural/biophysical factors which further need to be tested.

Thermal stability studies indicated that DRNase^®^ retained its native structure up to 40 °C, beyond which a progressive loss of secondary structure was observed. These features combined with excellent storage stability- maintaining full enzymatic activity after one year at -20 °C Benchmarking against commercially available nuclease confirmed DRNase^®^’s superior functional specificity and catalytic performance. Furthermore, minimal host cell lysis during fermentation contributed to the high purity of secreted protein, even before chromatography, illustrating an intrinsic advantage of extracellular secretion in reducing host-derived impurities.

In addition to DRNase^®^, another secreted endonuclease DNase I demonstrated RNase-free activity, efficiently degrading contaminating DNA while preserving RNA integrity. This is particularly valuable in mRNA vaccine manufacturing and molecular diagnostics, where selective DNA removal is critical [20, 21, 27]. These nucleases also exhibit significant therapeutic potential beyond their conventional applications. Notably, they have shown promise in treating respiratory diseases by breaking down extracellular DNA that contributes to mucus viscosity [26, 27]. Additionally, their ability to modulate inflammatory responses and promote tissue regeneration positions them as valuable agents in the management of chronic wound healing [25, 27, 37].

## Conclusions

This study establishes the BacSec^®^ secretion platform as a robust and versatile system for recombinant protein manufacturing, demonstrated here with DRNase^®^ and DNase I as representative models. By leveraging *E. coli* as a cost-efficient production host, BacSec^®^ circumvents the inherent bottlenecks of conventional intracellular expression systems, including toxic by-product accumulation, protein aggregation, limited dilution rates, and downstream complexity. The continuous, secretion-based architecture eliminates the need for cell disruption and refolding, streamlining purification and preserving native protein activity. Integration with perfusion-based fermentation further amplifies process performance, nearly doubling volumetric titer while minimizing metabolic inhibition. Although secretion of complex, disulfide-rich proteins remains a broader challenge, the BacSec^®^ framework provides a rational path forward through signal peptide engineering and host strain optimization. Importantly, the use of chemically defined media ensures process reproducibility, facilitates regulatory alignment, and supports seamless scale-up. Collectively, these advances position BacSec^®^ as a next-generation microbial production platform capable of delivering high-purity, biologically active proteins at industrial scale, with direct applicability across biopharmaceutical, diagnostic, and enzyme markets. By uniting secretion biology with continuous processing, BacSec^®^ has the potential to redefine microbial protein manufacturing and set a new standard for efficiency, scalability, and product quality in the bioeconomy.

## Materials and methods

### Reagents and materials

Most of the chemicals used in this study were of compendial grade and were purchased from Sigma-Millipore, with their respective CAS numbers provided in Table S3. *E. coli* codon-optimized genes were synthesized by Genscript (NJ, USA). Denarase^®^ from c-Lecta Benzonase^®^ from Merck-Millipore, and DNase I from Thermo Fisher. Protein markers included SeeBlue Plus from Invitrogen (USA), broad range OG-12 marker from Takara (India), Puregene broad range marker from Genetix (India), and broad range marker from Biocomma (China). LB broth was purchased from BD Biosciences; Kanamycin and IPTG from Sigma-Aldrich; Q-Sepharose FF from Cytiva;; and ET-Clean-S resin from JNC; Endotoxin detection kits from Hzymes, China. Nuclease A (nucA) from *Serratia marcescens* is commercially available with same amino acid sequence under various trade names, including Benzonase^®^ (Merck) [21], Denarase^®^ (c-LEcta) [38], DRNase^®^ (Oncosimis) and TurboNucleae^®^ (Accelagen). The DRNase^®^ protein sequence used in this study is identical at the amino acid level to a commercially available enzyme from Oncosimis Biotech Pvt Ltd.

### Plasmid and *E. coli* strain

The pBacSec plasmid was artificially synthesized as a medium-copy-number vector (GenScript, NJ, USA) and contains a T7-inducible promoter and terminator. A schematic of the plasmid map is shown in Figure S1A, and the complete plasmid sequence with the gene insert is provided in Table S1 [39]. For protein expression, the pBacSec plasmid carrying the gene of interest was transformed into the *E. coli* BL21 BOP2 strain. This in-house–developed strain is derived from BL21 (DE3) and functions as an IPTG-inducible secretory host [36]. The BOP2 strain was generated through sequential exposure to chemical mutagens and UV irradiation, resulting in multiple point mutations and deletions in genes associated with the secretory pathway (detailed characterization ongoing and thus data not disclosed here).

### Colony screening

Up to 24 colonies are picked from the transformation plate and individually inoculated into 3 mL of chemically defined medium in sterile culture tubes. These cultures were incubated at 37 °C in a shaker incubator set at 180 rpm until they reach an optical density (OD₆₀₀) of approximately 0.6. At this point, 200 µL of each culture was harvested and stored as glycerol stocks at -80 °C for future use. The remaining cultures were induced with 0.25 mM IPTG and incubated overnight (16 h) at 37 °C and 180 rpm. After induction, cultures typically reach an OD₆₀₀ of 1.2 to 1.5. Following induction, 75 µL of each culture was collected and centrifuged at 15,000 ×g for 5 min. The cultured media was carefully separated, and the pellet was resuspended in 75 µL of 1× PBS. For each 75 µL sample, 25 µL of 4× SDS loading dye was added. Both the resuspended pellet and cultured media samples are then heat-denatured at 95 °C for 10 min. A 12% SDS-PAGE gel was prepared, and 20 µL of each sample was loaded into the wells. Gel electrophoresis was performed at a constant voltage of 80–120 V for approximately one hour or until the dye front reaches the bottom of the gel. The cultured media fractions are analyzed to assess the secretion efficiency of the recombinant protein, while the pellet fractions are examined to confirm successful induction. Based on the SDS-PAGE results, selected positive clones are labeled and carried forward for further downstream processing.

### High-density fed-batch fermentation at 3 L scale

A 300 mL seed culture was prepared a day prior to fermentation by inoculating 2–3 µL (a small amount using a sterile 1 mL pipette tip) of a glycerol stock containing a high-expressing recombinant clone into chemically defined medium. The culture was incubated overnight at 37 °C with shaking at 180 rpm. By the next day, the seed culture typically reaches an OD₆₀₀ of 3–5. For fermentation, 300 mL of the seed culture was transferred into a 4 L fermenter vessel containing 3 L of freshly prepared OSB medium (as described in Table S2), along with 10 mL of kanamycin stock solution (50 mg/mL), maintaining a 1:10 inoculation ratio. The fermentation process was carried out under tightly controlled conditions: temperature at 37 °C, pH maintained at 6.85, and dissolved oxygen (DO) at 30%. A glucose feed strategy was used to support high cell density, with glucose and OD₆₀₀ monitored every hour. Feed media (50% glucose, 1X thiamine and 1X trace elements) was given to increase the biomass. The glucose concentration was estimated using a glucose meter with standard blood glucose test strips, which were sufficient for this purpose. Starting from an initial glucose concentration of 25 g/L, induction was initiated once the glucose level drops to approximately 10 g/L, typically after about 5 h of cultivation. At this point, 0.25 mM IPTG (from a 1 M stock solution) was added to induce protein expression. The culture generally reaches an OD₆₀₀ of 35–40 at the time of induction. A sample was taken from the fermenter at this stage for SDS-PAGE analysis to assess protein expression levels.

### Centrifugation, TFF, and FPLC chromatography

After fermentation, the culture was centrifuged at 15,000 ×g for 30 min at 4 °C. The cultured media (about 3 L) was collected and filtered using a 0.45 μm membrane filter. The filtered cultured media was then processed using Tangential Flow Filtration (TFF) with 300 kDa and 10 kDa cutoff cassettes. The 300 kDa permeate and the 10 kDa retentate, which contained the protein of interest (molecular weight > 10 kDa), were collected. The 10 kDa retentate was dialyzed using a buffer at pH 8 with a < 10 kDa PES cassette. About 3 dia-volumes of buffer were used. Dialysis was done at a flow rate of 100 mL/min and took around 25–30 min. The final dialyzed retentate (about 500 mL) was collected. Samples were taken after centrifugation, filtration, TFF, and dialysis (both retentate and permeate) and checked by SDS-PAGE to confirm the presence of the protein. The 500 mL retentate was then purified using a Q-Sepharose FF column or by Akta Purifier or Sepragen Quantasep FPLC. For Anion exchange chromatography, a binding buffer 25 mM Tris, 5% glycerol, pH 8 was used to allow the protein to bind to the column. Elution was done using an elution buffer (25 mM Tris, 1 M NaCl, 5% glycerol, pH 8), and the eluted protein was checked by SDS-PAGE. The protein-containing fractions were dialyzed again using a 10 kDa PES cassette and a buffer at pH 8.0 or 7.5 (25 mM Tris, 5% glycerol). This dialyzed sample was passed through a 25 mL ET-Clean-S resin column at 5 mL/min to remove endotoxins present in the samples. The final product was tested using a Qubit fluorometer to check for DNA and RNA contamination caused by possible cell lysis during fermentation.

### Perfusion-based fermentation with *E. coli* to secrete protein of interest into culture media [33, 40]

A single colony was picked and inoculated into 300 mL of chemically defined medium and grown overnight at 37 °C with shaking at 180 rpm. This seed culture was used to inoculate the perfusion bioreactor at a 1:10 ratio. The 4 L fermenter vessel (3 L working volume) was cleaned, assembled, and autoclaved. The medium was prepared and autoclaved separately or filtered as needed. After inoculation, the culture was grown in batch mode at 37 °C until the OD₆₀₀ reached 10–15. The pH was controlled at 6.8-7.0 using ammonia or NaOH, and dissolved oxygen (DO) was maintained above 30% saturation using air and oxygen sparging in cascade mode. After reaching the target OD, the culture was switched to perfusion mode using a hollow fiber module or TFF system with a 300 kDa membrane to retain cells. Fresh media was fed continuously, and the same volume of cell-free cultured media was removed to maintain a constant culture volume. The feed rate was adjusted based on glucose consumption and DO levels. Glucose levels were monitored hourly using a glucose meter. When glucose dropped below 10 g/L (starting from 25 g/L), feeding was adjusted to maintain optimal levels. IPTG was added at a final concentration of 0.25 mM to induce protein expression when OD₆₀₀ reached 10–15. The culture was maintained in perfusion mode for 15 h post-induction depending on the expression kinetics. Samples were collected at regular intervals for SDS-PAGE analysis to monitor expression and secretion. The culture was harvested either continuously through the permeate or by stopping the run and collecting the retentate. Cultured media and cell pellet fractions were analyzed to assess protein localization on 12% SDS-PAGE.

### DNA and RNA degradation assay

To assess the activity and specificity of recombinant DNase I, 1 µg of total RNA was mixed with 1 µg of genomic DNA to prepare a DNA–RNA substrate mixture (2 µg total nucleic acids). The mixture was incubated with varying concentrations of DNase I in a final reaction volume of 25 µL. After incubation at 37 °C for 10 min, the reactions were terminated, and samples were analyzed by electrophoresis on a 1% agarose gel. Nucleic acids were visualized under a UV transilluminator to evaluate degradation patterns.

### ELISA

In-house developed affinity-purified rabbit polyclonal antibodies against DRNase^®^ and DNase I were utilized to develop a standard sandwich ELISA for antigen detection in culture media. ELISA plates were initially coated with 25 µg/mL of antibody, prepared in 0.2 M sodium bicarbonate buffer (pH 9.7), and incubated overnight at room temperature to allow for efficient binding. Following coating, wells were washed once with 1X PBS to remove any unbound antibody. Standards samples were prepared in a series of 10-fold serial dilutions, ranging from 1000 ng/mL to 0.001 ng/mL in 1X PBS. A 1X PBS blank was included as a negative control. One hundred microliters of each antigen dilution and serial diluted culture media samples were added to the respective wells, and the plate was incubated for 2–3 h at room temperature on a shaker to facilitate antigen binding. After incubation, wells were washed twice with 1X PBS to remove unbound antigen. A secondary HRP-conjugated anti-rabbit IgG antibody diluted 1:1000 in 1X PBS, was added at 100 µL per well. The plate was incubated for 1 h at room temperature on a shaker. Post-incubation, wells were washed once with 0.2% Tween-20 in PBS followed by two washes with 1X PBS, each for 5 min, to minimize background noise. For detection, 100 µL of TMB substrate solution (1 mg/mL in 0.2 M sodium phosphate-citrate buffer, pH 5.0) was added to each well and allowed to react for approximately 15–20 min at room temperature until a visible blue color developed. The reaction was terminated by the addition of 50 µL of 2 N sulfuric acid (H₂SO₄), resulting in a yellow color. Absorbance was measured at 450 nm using a microplate reader.

### CD spectrometry

Circular dichroism spectroscopy was used to evaluate the secondary structure of the purified protein [41]. The protein sample was dialyzed extensively against 25mM Tris buffer (pH 8) to remove interfering salts and other UV-absorbing components. After dialysis, the protein concentration was adjusted to approximately 1 mg/mL, and the final concentration was confirmed using a UV spectrophotometer (A280) or BCA assay. The CD spectra were recorded using a Jasco J-810 spectropolarimeter (or equivalent) equipped with a temperature-controlled quartz cuvette of 0.1 cm path length. Spectra were collected over the far-UV region (190–260 nm) at 25 °C, with a bandwidth of 1 nm, a response time of 1 s, and a scanning speed of 50 nm/min. Each spectrum was obtained by averaging three accumulations to improve the signal-to-noise ratio. A buffer blank was recorded under identical conditions and subtracted from the sample spectra to correct for baseline effects.

### Circular dichroism (CD) thermal denaturation

Thermal unfolding of DRNase was monitored using circular dichroism (CD) spectroscopy. Near-UV CD spectra were recorded at [251 nm and 291 nm] with a [1 mm] quartz cuvette [41]. The temperature was increased from [e.g., 20 °C to 75 °C] at a rate of [e.g., 1 °C/min], and spectra were collected at each increment. To determine the melting temperature (Tm), ellipticity was tracked at a single wavelength that reflects the unfolding transition. The midpoint of the unfolding curve was defined as the Tm.

### Intact mass and peptide mapping by mass spectrometry

Intact mass analysis and peptide mapping were performed using high-resolution electrospray ionization mass spectrometry on a Thermo Fisher Ultimate 3000 UHPLC system coupled to a Q Exactive Plus Orbitrap HRMS system [42]. For intact mass analysis, purified protein samples were desalted and directly injected into the mass spectrometer to determine the molecular weight and confirm the expected mass and structural integrity. The analysis was conducted using a Hypersil Gold C8 column (100 mm × 2.1 mm, 5 μm) at 40 °C, with mobile phase A as 0.1% formic acid in water and mobile phase B as 100% acetonitrile. The instrument operated in positive polarity mode with a scanning range of 800–4000 m/z and a resolution of 140,000. For peptide mapping, the protein was enzymatically digested using trypsin or GluC, and the resulting peptides were separated using reverse-phase chromatography on a Hypersil C18 column (100 mm × 2.1 mm, 1.9 μm) at 40 °C. The mobile phase A was 0.1% formic acid in water, and mobile phase B consisted of 80:20:0.1 (acetonitrile: water: formic acid). MS/MS analysis was performed in positive polarity with a scanning range of 300–2000 m/z and a resolution of 70,000/17,500. The acquired spectra were matched against theoretical peptide sequences to assess identity, sequence coverage, and any post-translational modifications or sequence variants. Together, intact mass and peptide mapping provided comprehensive structural and quality characterization of the protein.

### Purity analysis by UPLC using the C18 column

Protein purity was assessed using a Waters UPLC H-Bio system equipped with a reverse-phase C18 column (1.7 μm, 2.1 × 100 mm). The analysis was performed at a column temperature of 40 °C with a flow rate of 0.5 mL/min. A linear gradient from 5% to 80% acetonitrile containing 0.1% trifluoroacetic acid (TFA) was applied over 12 min, followed by a wash and re-equilibration step. Mobile phase A consisted of water with 0.1% TFA, and mobile phase B consisted of acetonitrile with 0.1% TFA. Protein samples were diluted in water or 0.1% TFA and filtered through a 0.22 μm syringe filter prior to injection. Detection was carried out at 214 nm. Purity was determined by calculating the percentage area of the main peak relative to the total chromatographic area. A single dominant peak with minimal additional peaks indicated high purity.

### Enzyme activity assay

DRNase^®^ endonuclease unit definition- One unit of DRNase^®^ Nuclease was defined as the amount of enzyme that causes an ∆A260 of 1.0 in 30 min (salmon sperm DNA into acid-soluble nucleotides), which corresponds to complete digestion of ~ 50 µg of DNA in buffer containing 25 mM Tris-HCl buffer (pH 8.0), 20mM NaCl, 2 mM MgCl_2_ [21]. DNase I endonuclease unit definition- One unit of enzyme activity is defined as the amount of enzyme required to completely degrade 1 µg of plasmid DNA within 10 min at 37 °C in DNase I reaction buffer (10 mM Tris-HCl, 2.5 mM MgCl₂, 0.5 mM CaCl₂, pH 7.5) [43]. DRNase^®^ shows 1.5-1.6 × 10^6^ units per milligram of purified protein whereas DNase I shows >60,000 units per milligram of purified protein.

### Endotoxin analysis

Quantitative endotoxin levels were measured using a recombinant Factor C–based endotoxin detection kit (Hzymes Biotechnology, China), following the manufacturer’s instructions. In addition, qualitative analysis was performed using the Limulus Amebocyte Lysate (LAL) gel clot assay to confirm the absence of detectable endotoxin contamination.

### Statistical analysis

Statistical analysis was performed using Student’s *t*-test in Excel sheet. All values are expressed as mean ± SEM.

## Supplementary Information

Below is the link to the electronic supplementary material.

Supplementary Material 1

## Data Availability

All data generated or analyzed during this study are included in the main text and/or the supplementary information files accompanying this manuscript. Plasmids and bacterial strains used in this study are available upon reasonable request and are subject to the terms of the Uniform Biological Material Transfer Agreement (UBMTA). Requests for these materials should be directed to the authorized representative of Oncosimis Biotech Private Limited.

## Abbreviations

BacSec®: Bacterial Secretion
nucA: Nuclease A
AI: After Induction
ATP: Adenosine Triphosphate
HlyA: Alpha Hemolysin
T1SS: Type 1 Secretory System
ELISA: Enzyme-Linked Immunosorbent Assay
SDS-PAGE: Sodium Dodecyl Sulfate Polyacrylamide Gel Electrophoresis
CD: Circular Dichroism
IPTG: Isopropyl β-D-1-thiogalactopyranoside
RNA: Ribonucleic Acid
TFF: Tangential Flow Filtration
UPLC: Ultra Performance Liquid Chromatography
OD: Optical Density
DO: Dissolved Oxygen
