# Supplementary material for: Continuous secretory production in E. coli enables scalable, high-titer manufacturing of active recombinant endonucleases
Source: J Biol Eng. 2025 Dec 15;20:8. doi: 10.1186/s13036-025-00590-0 (PMC12822159; doi:10.1186/s13036-025-00590-0)
Supplement: Supplementary file 1 — Supplementary Material 1 [file 13036_2025_590_MOESM1_ESM.docx]

**Continuous secretory production in *E. coli* enables scalable, high-yield manufacturing of active recombinant endonucleases**

Sudarsana Reddy **Lokireddy^1,2^;** Chennakesavulu **Thummadhi^1^,** Pratyusha **Godavarty^1,2^;** Venkateswarlu **Petla^1^;** Akhila **Munimanda^1^;** Sridhar Rao **Kunchala^1^**^#^**;** Ramakrishna **Vadde**^2#^

**Supplemental Figures, Legends and Tables**

**Fig. S1 Optimal signal peptide for secretion**

**A**. BacSec® plasmid map with DRNase® insert. Whole plasmid sequence provide in Table-S1. B. The *nucA* gene, codon-optimized for *E. coli*, was synthetically constructed with various signal peptides (yefB, modified yefB, LPP, pelB, ompA, and ompF) and cloned into the pBacSec plasmid. Constructs were transformed into the secretion-optimized *E. coli* strain BOP2. Expression and extracellular secretion were assessed by SDS-PAGE following IPTG induction in 3 mL chemically defined cultures. **C.** Anion exchange chromatography profile of DRNase® purification following the tangential flow filtration (TFF) step. AI = After Induction

**Fig. S2 DRNase® exhibits Protease-free superior activity, Stability**

**A & B.** Digestion of 50 µg salmon sperm DNA with 1 unit of DRNase® in 25 mM Tris-HCl buffer containing 20 mM NaCl and 2 mM MgCl₂, evaluated using the acid-soluble nucleotide assay across a pH range (2–10) and after multiple freeze-thaw cycles. Experiments were repeated at least three times. Error bar represents the SEM. **C.**  Increasing concentrations of DRNase® were incubated with 10 µg BSA for 1 hour at 37 °C. SDS-PAGE was performed to evaluate BSA degradation. **D.** Agarose gel analysis (1%) of DNA digestion after 20-minute incubation with 1 or 0.5 IU of DRNase® under standard buffer conditions. Lane 1 & 2: room temperature-stored samples; 3 & 4: 4 °C-stored samples; 5 & 6: −20 °C-stored samples; 7: no enzyme control; 8: DNA ladder. E. Densitometry data of Figure-2C and 2D represented in bar graph.

**Fig. S3 Intact Mass Analysis of DRNase®**

**A.** UHPLC-UV-MS chromatogram showing the intact mass profile of purified DRNase®. **B.** Zoomed-in deconvoluted mass spectrum confirming the molecular weight and homogeneity of the DRNase® protein.

**Fig. S4 Secondary Structure and Thermal Stability of DRNase® Assessed by Circular Dichroism (CD) Spectroscopy**

**A.** Far-UV CD spectra recorded at 25 °C indicate that DRNase® comprises 31.9% α-helix, 13.7% β-sheet, 14.5% turns, and 39.9% unordered structures. **B.** Near-UV CD spectra were used to assess thermal structural stability from 25 °C to 75 °C in 5 °C increments. Minimal structural changes were observed up to 40 °C, indicating good thermal stability in this range.

**Table S1 Signal peptides and Protein sequences used in this manuscript**

**Table S2 Chemical Defined Medium**

**Table S3 List of Chemicals and their CAS numbers**
